# Novel role of LLGL2 silencing in autophagy: reversing epithelial-mesenchymal transition in prostate cancer

**DOI:** 10.1186/s40659-024-00499-w

**Published:** 2024-05-08

**Authors:** Geum-Lan Hong, Kyung-Hyun Kim, Yae-Ji Kim, Hui-Ju Lee, Sung-Pil Cho, Seung-Yun Han, Seung Woo Yang, Jong-Soo Lee, Shin-Kwang Kang, Jae-Sung Lim, Ju-Young Jung

**Affiliations:** 1 Department of Veterinary Medicine, Institute of Veterinary Science, College of Veterinary Medicine, Chungnam National University, 99 Daehak-ro, Yusung-gu, Daejeon, 34134 Republic of Korea; 2 Department of Anatomy, College of Medicine, Konyang University, Daejeon, 35365 Republic of Korea; 3 Department of Thoracic and Cardiovascular Surgery, College of Medicine, Chungnam National University Hospital, Daejeon, 35015 Republic of Korea; 4 Department of Urology, College of Medicine, Chungnam National University Hospital, Daejeon, 35015 Republic of Korea

**Keywords:** Autophagy, EMT, LLGL2, Proliferation, Prostate cancer

## Abstract

**Purpose:**

Prostate cancer (PCa) is a major urological disease that is associated with significant morbidity and mortality in men. LLGL2 is the mammalian homolog of Lgl. It acts as a tumor suppressor in breast and hepatic cancer. However, the role of LLGL2 and the underlying mechanisms in PCa have not yet been elucidated. Here, we investigate the role of LLGL2 in the regulation of epithelial-mesenchymal transition (EMT) in PCa through autophagy in vitro and in vivo.

**Methods:**

PC3 cells were transfected with siLLGL2 or plasmid LLGL2 and autophagy was examined. Invasion, migration, and wound healing were assessed in PC3 cells under autophagy regulation. Tumor growth was evaluated using a shLLGL2 xenograft mouse model.

**Results:**

In patients with PCa, LLGL2 levels were higher with defective autophagy and increased EMT. Our results showed that the knockdown of LLGL2 induced autophagy flux by upregulating Vps34 and ATG14L. LLGL2 knockdown inhibits EMT by upregulating E-cadherin and downregulating fibronectin and α-SMA. The pharmacological activation of autophagy by rapamycin suppressed EMT, and these effects were reversed by 3-methyladenine treatment. Interestingly, in a shLLGL2 xenograft mouse model, tumor size and EMT were decreased, which were improved by autophagy induction and worsened by autophagy inhibition.

**Conclusion:**

Defective expression of LLGL2 leads to attenuation of EMT due to the upregulation of autophagy flux in PCa. Our results suggest that LLGL2 is a novel target for alleviating PCa via the regulation of autophagy.

**Supplementary Information:**

The online version contains supplementary material available at 10.1186/s40659-024-00499-w.

## Introduction

Lethal giant larvae (Lgl) is associated with epithelial apical-basal polarity machinery and was first identified in Drosophila. Lgl regulates self-renewal and differentiation of progenitor cells ascribed to tumor suppressor [1–3]. Several studies have revealed that dysregulation of Lgl is closely associated with tumor development and progression. There are two human orthologs Lgl, LLGL1 and LLGL2. Among them, LLGL2 expression is associated with cancer progression. Recently, Saito et al. showed that LLGL2 expression in estrogen receptor (ER) + breast cancer is remarkably higher than that in ER- breast cancer tissue and that LLGL2 promotes cell proliferation under nutrient stress in ER + breast cancer [4]. In addition, LLGL2 can promote cell proliferation in hepatocellular carcinoma by activating phosphatidylinositol 3-kinase (PI3K)/Akt signaling and may serve as a therapeutic target in hepatocellular carcinoma [5]. However, the molecular mechanisms underlying the role of LLGL2 in prostate cancer (PCa) progression remain unknown.

PCa is a major urological disease related to significant morbidity and mortality in men [6]. It is the second most common cause of cancer-related death in the male population. Despite the rapid development of various therapies for PCa, the 5-year survival rate of late-stage PCa patients is only 29% in the United States [7]. This is mainly because PCa progression is highly aggressive and is prone to metastasis. Therefore, understanding the molecular mechanisms underlying PCa progression is urgently required for the development of new and effective therapeutic interventions and treatments for PCa.

Epithelial-mesenchymal transition (EMT) is a significant event that regulates the initial steps of cancer progression and metastasis in various cancers, including PCa [8]. During EMT, cancer cells lose their epithelial characteristics and acquire mesenchymal, migratory, and invasive phenotype [8]. This condition is closely associated with poor clinical outcomes and resistance to treatment in various cancers [9]. Similar to other tumor types, recent studies have emphasized the role of EMT in PCa, progression and metastasis [10]. Destruction of the epithelial phenotype indicates the impairment of epithelial homeostasis. Autophagy, conserved metabolic process that maintains cellular homeostasis, is considered an ideal mechanism for PCa treatment [11]. It not only influences cell proliferation but can also affect EMT in PCa [12]. We recently showed that silencing LLGL2 can induce autophagy and lead to the suppression of BPH-1 prostate epithelial cell proliferation [13]. However, the functions and underlying mechanisms of LLGL2 in autophagy and tumor progression in PCa remain unknown. Therefore, the objective of this study was to determine the expression of LLGL2 in PCa and to explore the role of LLGL2 in EMT in PCa through autophagy, both in vitro and in vivo.

## Materials & methods

### Patient specimens

Non-cancerous benign prostatic hyperplasia; BPH (*n* = 5) and PCa tissues (*n* = 5) were collected from the Chungnam National University Hospital. Informed consent was obtained before obtaining the human tissues. This study was approved by the Institutional Review Board of the Chungnam National University (202,207-BR-096-01). The details of the samples are provided in Supplementary Table 1.

### Cell culture and reagents

PC3 cells were purchased from American Type Culture Collection (ATCC, Rockville, MA, USA). RPMI 1640 supplemented with 10% fetal bovine serum, and 1% penicillin/streptomycin (Gibco, Waltham, MA, USA) was used for PC3 cell culture. The culture medium was passaged every 3 days. Rapamycin (Rapa) and 3-methyladenine (3MA) were purchased from Sigma Aldrich (St. Louis, MO, USA).

### siRNA and plasmid transfection assay

The sequence of LLGL2 siRNA (siLLGL2) was as follows: 5′-AATCGCTTTGCAAGGAAAGGG-3′ - siLLGL2 was transfected into PC3 cells using FuGENE® SI Transfection Reagent (Fugene LLC, Middleton, WI, USA) according to manufacturer’s instructions. Detailed experimental methods are provided in Supplementary Materials and Methods. To overexpress LLGL2, *LLGL2* was subcloned into the Strep-tagged pEXPR-IBA105 plasmid. PC3 cells were transfected with the LLGL2 overexpression plasmid (pLLGL2) using Lipofectamine 2000 reagent (Thermo Fisher Scientific, Waltham, MA, USA) according to manufacturer’s instructions. The efficiency of LLGL2 overexpression was evaluated by western blotting.

### Western blot analysis

PC3 cells and xenografted tissues were lysed using radio-immunoprecipitation assay buffer (Cell Signaling Technology, Danvers, MA, USA). Western blotting was performed as previously described [14]. For detailed experimental methods, see Supplementary Materials and Methods.

### Detection of autophagy flux

Autophagosome formation in PC3 cells was evaluated using the Premo™ Autophagy Tandem Sensor RFP-GFP-LC3B Kit (Thermo Fisher Scientific), according to the manufacturer’s instructions. PC3 cells were grown on eight well cell culture slides (SPL Life Science, Pocheon-si, Gyeonggi-do, Korea) and incubated with 6 µL of BacMam Reagent containing RFP-GFP-LC3B, overnight. On the subsequent day, PC3 cells were treated with siNC (negative control siRNA), siLLGL2, pNC (negative control plasmid), or pLLGL2 for 48 h. Nuclei were counterstained with 4’,6-diamidino-2-phenylindole (DAPI, Vector Laboratories). Fluorescence was examined using a Zeiss LSM 880 microscope with an Airyscan confocal microscope (Carl Zeiss, Jena, Germany) at wavelengths of 488 and 592 nm. Images were captured using ZEN software.

### Cell viability assays

PC3 cells were seeded into 96-well plates (5 × 10^3^ cells/well). PC3 cells were treated with siNC, siLLGL2, pNC, or pLLGL2. After 48 h, cell proliferation was measured using EZ-Cytox Cell Viability Assay Kit (Biomax, Seoul, Korea). Ten µL of EZ-Cytox working solution per 100 µL of medium were added to each well of a 96-well plate. The cells were incubated for 1 h at 37 °C. A microplate reader (INNO, LTek, Gyeonggi-do, Korea) was used to measure absorbance.

### Cell migration and invasion assay

Transwell migration assays (Corning, NY, USA) were performed for the migration and invasion of cells using Transwell chambers (Corning, NY, USA). For detailed experimental methods, see Supplementary Materials and Methods.

### Wound healing assay

PC3 cells were seeded in 6 well plates at a density of 3 × 10^5^ cells/ well. A wound track was created on each plate by using a plastic scraper. After 24 h of culture, the wound areas were examined by using an Olympus microscope. All experiments were repeated three times. The migration rate was calculated.

### Immunofluorescence staining

PC3 cells were fixed with 4% paraformaldehyde for 10 min and blocked with 3% bovine serum albumin for 30 min at room temperature. Immunofluorescence staining was performed as previously described [14]. For detailed experimental methods, see Supplementary Materials and Methods.

### Generation of stable LLGL2 knockdown cell lines

For LLGL2 gene silencing, human LLGL2-shRNA sequences (5′-AATCGCTTTGCAAGGAAAGGG-3) were cloned into the lentiviral pLKO vector. The shRNA containing pLKO vector was packaged into high-titer lentivirus at the IBS Virus Facility (Daejeon, Republic of Korea). To calculate the knockdown efficiency, the virus was diluted with RPMI complete medium. Then 5 × 10^5^ wild-type PC3 cells were seeded into each well of 6-well plates and stably transfected with pLKO-LLGL2-shRNA-puro in the presence of polybrene (10 µg/mL, Thermo Fisher Scientific). After incubation at 37 ℃ for 72 h, the medium was replaced with a complete medium containing puromycin (Gibco) to select a stable cell pool.

### In vivo xenograft animal model

Animal studies were conducted in accordance with the International Animal Ethics Committee of Chungnam National University (202,103 A-CNU-039). Four-week-old male nude BALB/c mice were purchased from Orient Bio (Gyeonggi-do, South Korea). The animals were acclimated under stable conditions (22 ± 2 ℃, 55 ± 5% humidity) for one week. The animals were fed a standard diet and water ad libitum in a specific pathogen free animal facility. For detailed experimental methods, see Supplementary Materials and Methods.

### Immunohistochemistry

Mouse xenograft tissues and human tissues were fixed with 10% buffered formalin phosphate solution and embedded in paraffin as previously described [14]. For detailed experimental methods, see Supplementary Materials and Methods.

### Statistical analysis

All experiments were conducted in a double -blind manner. Results were randomly selected and were expressed as mean ± standard deviation (SD). GraphPad Prism (GraphPad Software, La Jolla, CA, USA) was used for all data analysis. The Mann-Whitney U test for non-parametric data was used to assess differences between two groups. For pairwise comparisons, one-way ANOVA was performed followed by a Tukey post hoc test when relevant. Statistical significance was set at *p* < 0.05.

## Results

### LLGL2 was overexpressed in PCa tissues

**Fig. 1:**
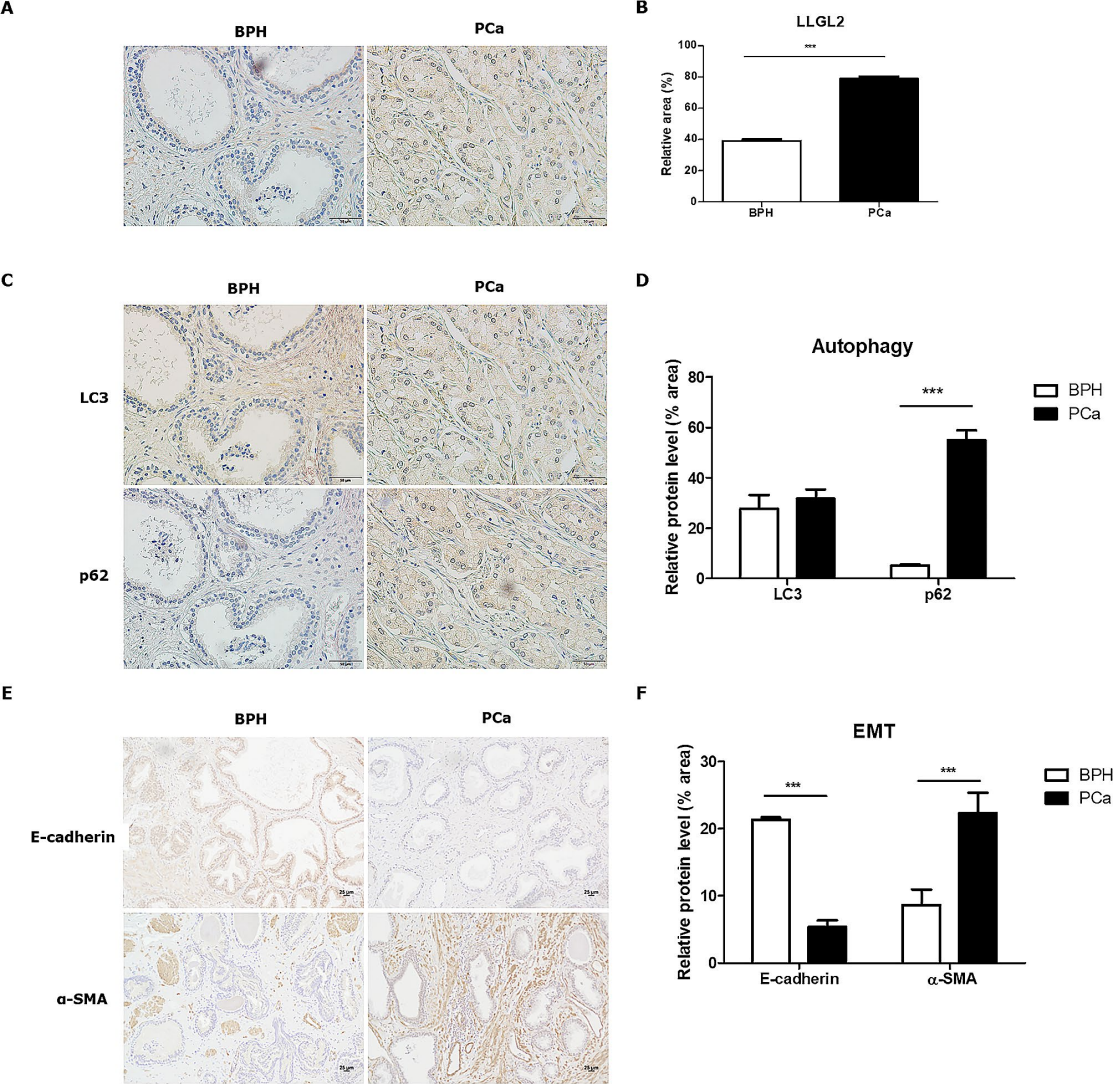
Expression of LLGL2 was higher in PCa tissues with downregulated autophagy and upregulated EMT (*n* = 5/ group). (**A**) Immunohistochemical staining of LLGL2 in the prostates of patients with non-cancer (BPH) and PCa patients. (**B**) Quantitative analysis of the positive areas in BPH and PCa tissues. (**C**) Immunohistochemical staining of LC3 and p62 in patients with BPH and PCa and quantitative analysis of the positive area (**D**). (**E**) Immunohistochemical staining of E-cadherin and α-SMA in patients with BPH and PCa and quantitative analysis of the positive area. The relative % area of positive densities was analyzed using Image J software. E) Immunostaining of E-cadherin and α-SMA in patients with BPH and PCa and quantitative analysis of positive area (F). Scale bar = 25 μm. mean ± SD. ^***^p < 0.001

To explore the expression of LLGL2 in PCa tissues, LLGL2 levels in non-cancerous prostate tissues (BPH) and PCa tissues were analyzed. As shown in Fig. 1A and B, LLGL2 was expressed in prostate epithelial cells, and its expression levels being significantly (*p* < 0.001) increased by 2-fold in PCa tissues (Fig. 1A and B). Kaplan-Maier assessment showed that overall survival rate was lower in PCa patients with overexpressed expression of LLGL2 protein than in patients with lower expression of LLGL2 protein (S1). Next, the levels of autophagy markers LC3B and p62 in BPH and PCa prostate tissues were detected using IHC. The positive immunostaining sites of LC3B was mainly in the cytoplasm and cell membranes of BPH and PCa tissues. Although no significant difference in LC3B protein levels was found between PCa and BPH tissues, the level of p62 protein was significantly higher in PCa tissues than in BPH tissues, indicating lower autophagy in PCa tissues (Fig. 1C and D). Furthermore, we analyzed the expression of the EMT-related markers, E-cadherin and α-SMA. E-cadherin was significantly reduced (*p* < 0. 001) and α-SMA was significantly increased (*p* < 0.001) in PCa tissues compared to BPH tissues (Fig. 1E and F). These results showed that LLGL2 expression was higher in PCa cells with downregulated autophagy and upregulated EMT.

### LLGL2 regulated autophagy flux in PC3 cells

**Fig. 2:**
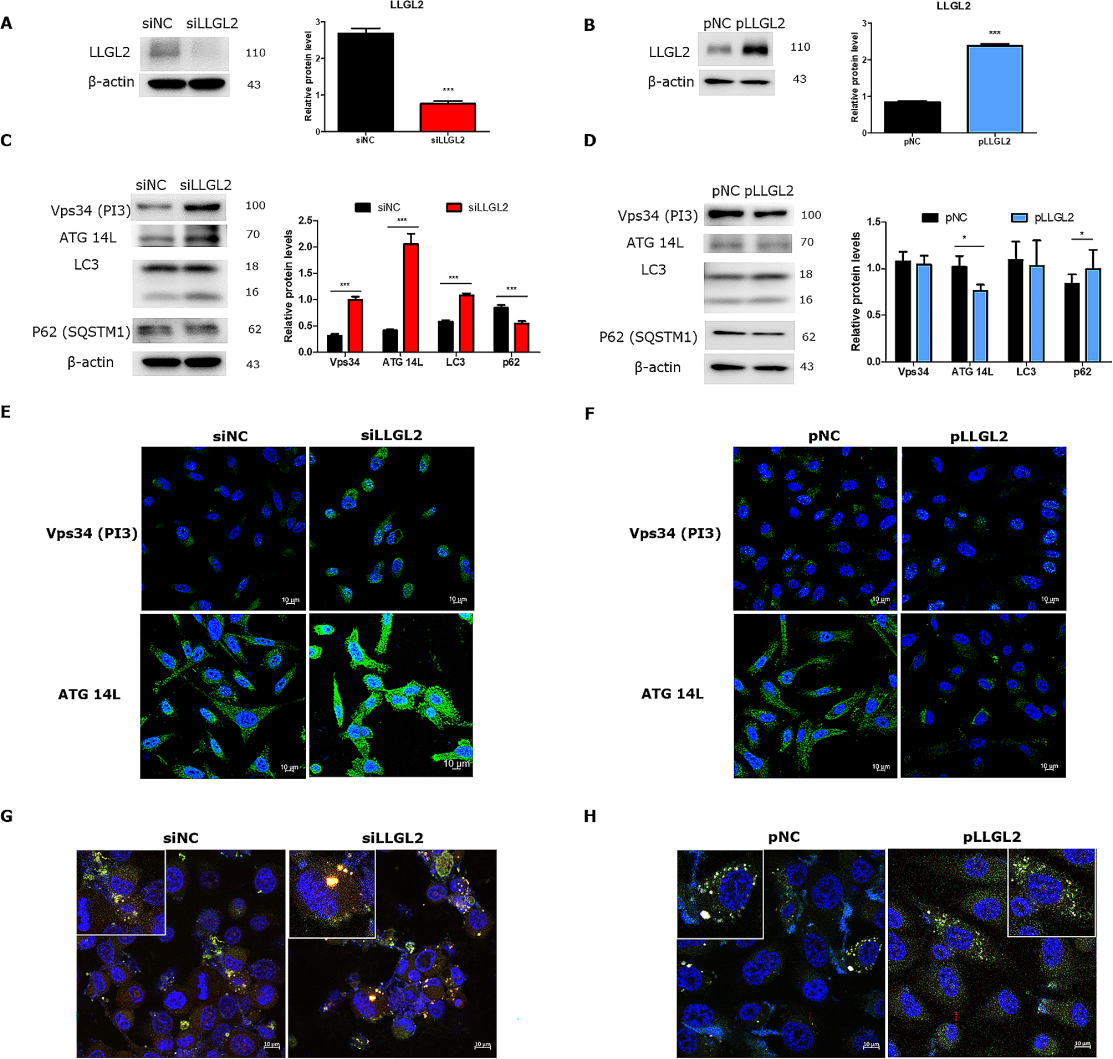
Silencing LLGL2 induced autophagy flux in PC3 cells. (**A**) Protein level of LLGL2 in PC3 cells transfected with siRNA sequences of LLGL2 (siLLGL2) or negative control (siNC) for 48 h as determined by western blotting and relative band intensities (right). (**B**) The protein level of LLGL2 in PC3 cells transfected with LLGL2 plasmid (pLLGL2) or control plasmid (pNC) for 48 h as determined by western blotting and relative band intensities (right, *n* = 3). (**C**) Protein levels of Vps34, ATG14L, LC3B and p62 were examined by western blotting in PC3 cells after transfection with siLLGL2 or siNC (left). Relative band intensities were analyzed using CS Analyzer 4 (*n* = 3, right). (**D**) The protein levels of Vps34, ATG14L, LC3B and p62 were examined using western blotting in PC3 cells after transfection with pLLGL2 or pNC (Left). Relative band intensities analyzed by CSAnalyzer 4 (*n* = 3, Right). (**E**) Representative immunofluorescence images of Vps34 or ATG14L in PC3 cells after transfection with siLLGL2 or siNC. (**F**) Representative immunofluorescence images of Vps34 or ATG14L in PC3 cells after transfection with pLLGL2 or pNC. (**G**) Analysis of GFP-RFP-LC3 fluorescent signals in PC3 cells after transfection with siLLGL2 or siNC and transient transfection with GFP-RFP-LC3 plasmid. (**H**) Analysis of GFP-RFP-LC3 fluorescent signals in PC3 cells after transfected with pLLGL2 or pNC and transiently transfected with GFP-RFP-LC3 plasmid. Scale Bar = 10 μm. Error bars, mean ± SD. ^*^p < 0.05 and ^***^p < 0.001, vs. control

To explore the role of LLGL2 in PC3 cells, we knocked down LLGL2 through siRNA transfection with siLLGL2 or overexpressing LLGL2 using pLLGL2. We knocked down LLGL2 using siRNA, and two interference sequences were used to evaluate the knockdown efficiency of LLGL2. Of these, siLLGL2-1 effectively suppressed LLGL2 expression in PC3 cells (Supplementary 2). Therefore, siLLGL2-1 was selected for subsequent experiments. To confirm the transfection of siLLGL2 and pLLGL2, the expression of LLGL2 in transfected-PC3 cells was verified by western blotting (Fig. 2A and B). The expression levels of Vps34, ATG 14 L, and LC3 were significantly (*p* < 0.01 and 0.001, respectively) increased, whereas the expression level of p62 was significantly reduced (*p* < 0.001) in siLLGL2 cells compared to that in siNC cells (Fig. 2C). Fluorescent staining also showed an increase in the number of Vps34 and ATG 14 L siLLGL2 cells (Fig. 2E). In contrast, the expression of ATG14L was significantly reduced and that of p62 was increased in pLLGL2 cells compared to that in pNC cells (Fig. 2D and F). Furthermore, we investigated autophagic flux using LC3 puncta (Fig. 2G and H). In siLLGL2 cells, yellow fluorescence by merging GFP-LC3 and RFP-lysosome increased, indicating the activation of autophagic flux. In contrast, the fluorescence was still green and the intensity was decreased in pLLGL2 cells compared to that in pNC cells, indicating that autophagy flux was reduced. These results indicated that silencing LLGL2 could activate autophagic flux in PC3 cells.

### LLGL2 regulated EMT of PC3 cells

**Fig. 3:**
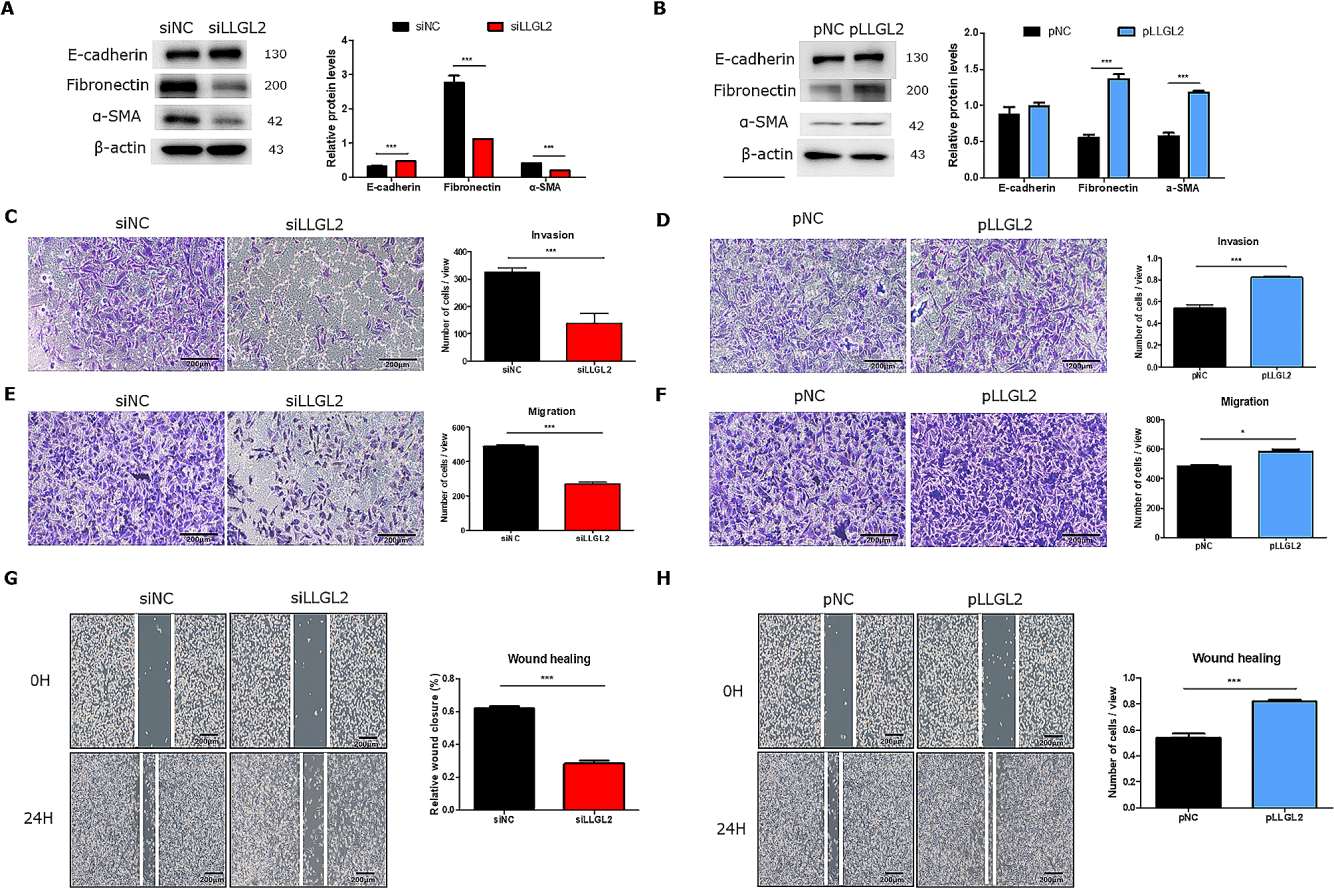
Silencing LLGL2 prevents EMT and overexpression of LLGL2 promotes EMT in PC3 cells. (**A**) Protein levels of E-cadherin, fibronectin, and α-SMA were examined by western blotting in PC3 cells after transfection with siLLGL2 or siNC (left). Relative band intensities were analyzed using CS Analyzer 4 (*n* = 3, right). (**B**) Protein levels of E-cadherin, fibronectin, and α-SMA were examined using western blotting in PC3 cells after transfection with pLLGL2 or pNC (left). Relative band intensities were analyzed using CS Analyzer 4 (*n* = 3, right). The invasion (*n* = 3, **C** and **D**) and migration (*n* = 3, **E** and **F**) assays were performed in PC3 cells after transfection with siLLGL2 or pLLGL2 (left). The number of cells in view was calculated using Image J (right). The wound healing assay (*n* = 3, **G** and **H**) was performed on PC3 cells after transfection with siLLGL2 or pLLGL2 (left). The relative % of wound closure was calculated using Image J (right). Scale Bar = 200 μm. Error bars, mean ± SD. ^*^p < 0.05 and ^***^p < 0.001, vs. control

To investigate the relationship between LLGL2 and EMT in PC3 cells, the protein levels of epithelial marker (E-cadherin) and mesenchymal markers (fibronectin, and α-SMA) were evaluated under LLGL2 regulation. When LLGL2 was knocked down, the expression of E-cadherin was significantly increased (*p* < 0.001), whereas the expression levels of mesenchymal markers were significantly decreased (*p* < 0.05 and 0.001) compared to those in siNC cells (Fig. 3A). In contrast, overexpression of LLGL2 decreased the expression of epithelial marker but significantly increased (*p* < 0.001) expression levels of the mesenchymal markers (Fig. 3B). Invasion, wound healing, and migration assays were performed to examine the metastatic ability of LLGL2 in PC3 cells. In the invasion assay, we observed that LLG2 knockdown decreased the invasion ability of PC3 cells by 50% compared to that of siNC cells (Fig. 3C). Furthermore, siLLGL2 reduced cell migration through the membrane and wound closure compared to siNC (Fig. 3E and G). In contrast, pLLGL2 showed enhanced invasion, earlier wound closure (*p* < 0.001), and significantly increased cell migration (*p* < 0.05, Fig. 3D and F, and 3H). These results indicated that LLGL2 silencing could inhibit EMT, resulting in reduced invasion, migration, and wound healing in prostate cancer cells.

### Silencing of LLGL2 prevented EMT through autophagy in PC3 cells

**Fig. 4:**
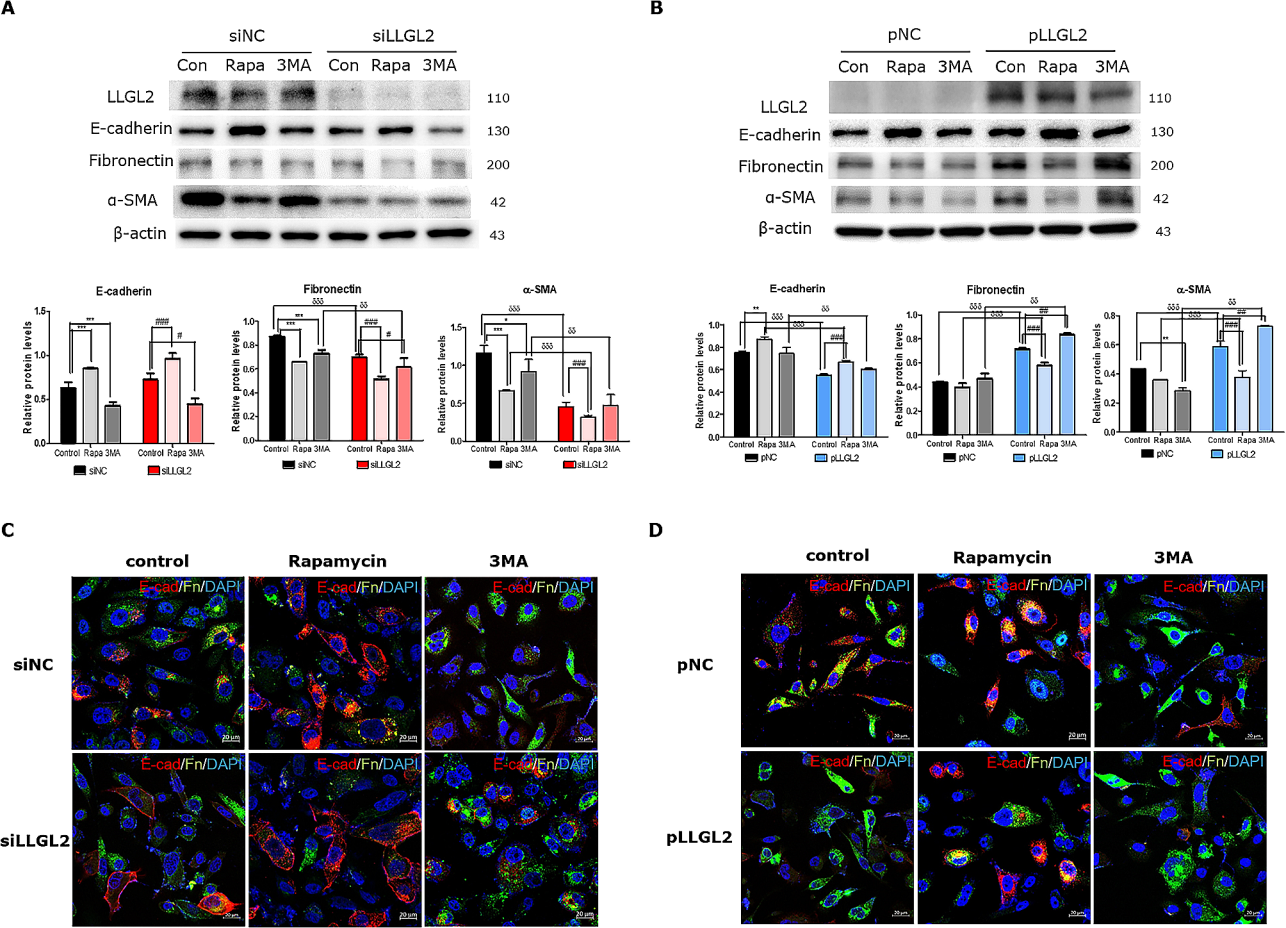
Silencing LLGL2 prevents EMT through autophagy. (**A**) Protein levels of E-cadherin, fibronectin, and α-SMA were examined using western blotting in PC3 cells after transfection with siLLGL2 or siNC for 48 h with rapamycin (Rapa, 100 nM) or 3-methyladenine (3MA, 5 mM, Top). Relative band intensities were analyzed using CS Analyzer 4 (*n* = 3, bottom). (**B**) The protein levels of E-cadherin, fibronectin, and α-SMA were examined using western blotting in PC3 cells after transfection with pLLGL2 or pNC for 48 h with rapamycin (Rapa, 100 nM) or 3-methyladenine (3MA, 5 mM), (Top). Relative band intensities were analyzed using CS Analyzer 4 (*n* = 3, Bottom). (**C**) Immunofluorescence staining of E-cadherin (red) and fibronectin (green) in PC3 cells transfected with siLLGL2 or siNC for 48 h with rapamycin (Rapa, 100 nM) or 3-methyladenine (3MA, 5 mM). (**D**) Immunofluorescence staining of E-cadherin (red) and fibronectin (green) in PC3 cells transfected with pLLGL2 or pNC for 48 h with rapamycin (Rapa, 100 nM) or 3-methyladenine (3MA, 5 mM). Scale Bar = 20 μm. Error bars, mean ± SD. ^*^p < 0.05, ^**^p < 0.01, ^***^p < 0.001, vs. siNC control, ^#^*p* < 0.05, ^##^*p* < 0.01, ^###^*p* < 0.001 vs. siLLGL2 control, and ^δ^*p* < 0.05, ^δδ^*p* < 0.01, and ^δδδ^*p* < 0.001 vs. respective control

We further explored EMT progression through induction or inhibition of autophagy using Rapa (an autophagy inducer) or 3MA (an autophagy initiation inhibitor). After the activation of autophagy by Rapa in PC3 cells, the expression of epithelial marker was significantly increased (*p* < 0.001) whereas as expression levels of the two mesenchymal markers were significantly reduced (*p* < 0.001). In contrast, 3MA treatment significantly downregulated the expression of epithelial marker and upregulated that of mesenchymal markers. These results imply that the activation of autophagy prevents EMT and that the inhibition of autophagy aggravate EMT in PC3 cells. Furthermore, siLLGL2 prevented EMT by increasing epithelial marker and reducing mesenchymal markers, which improved upon Rapa treatment and worsened upon 3MA treatment (Fig. 4A). Confocal images also showed autophagy flux, in which silencing of LLGL2 led to an increase in the E-cadherin (red fluorescence) in the plasma membrane and a weakening of fibronectin (green fluorescence), which improved with Rapa treatment but worsened with 3MA treatment (Fig. 4C). Although EMT is regulated by autophagy, the prevention of EMT caused by Rapa treatment was not as restored as that in pNC-Con cells during pLLGL2 treatment. In pLLGL2-3MA cells, EMT was exacerbated compared to that in pLLGL2-Con cells (Fig. 4B and D). Collectively, these results indicate that silencing LLGL2 prevent EMT through autophagic flux.

### Knockdown of LLGL2 inhibited tumor growth and EMT through autophagy in vivo

**Fig. 5:**
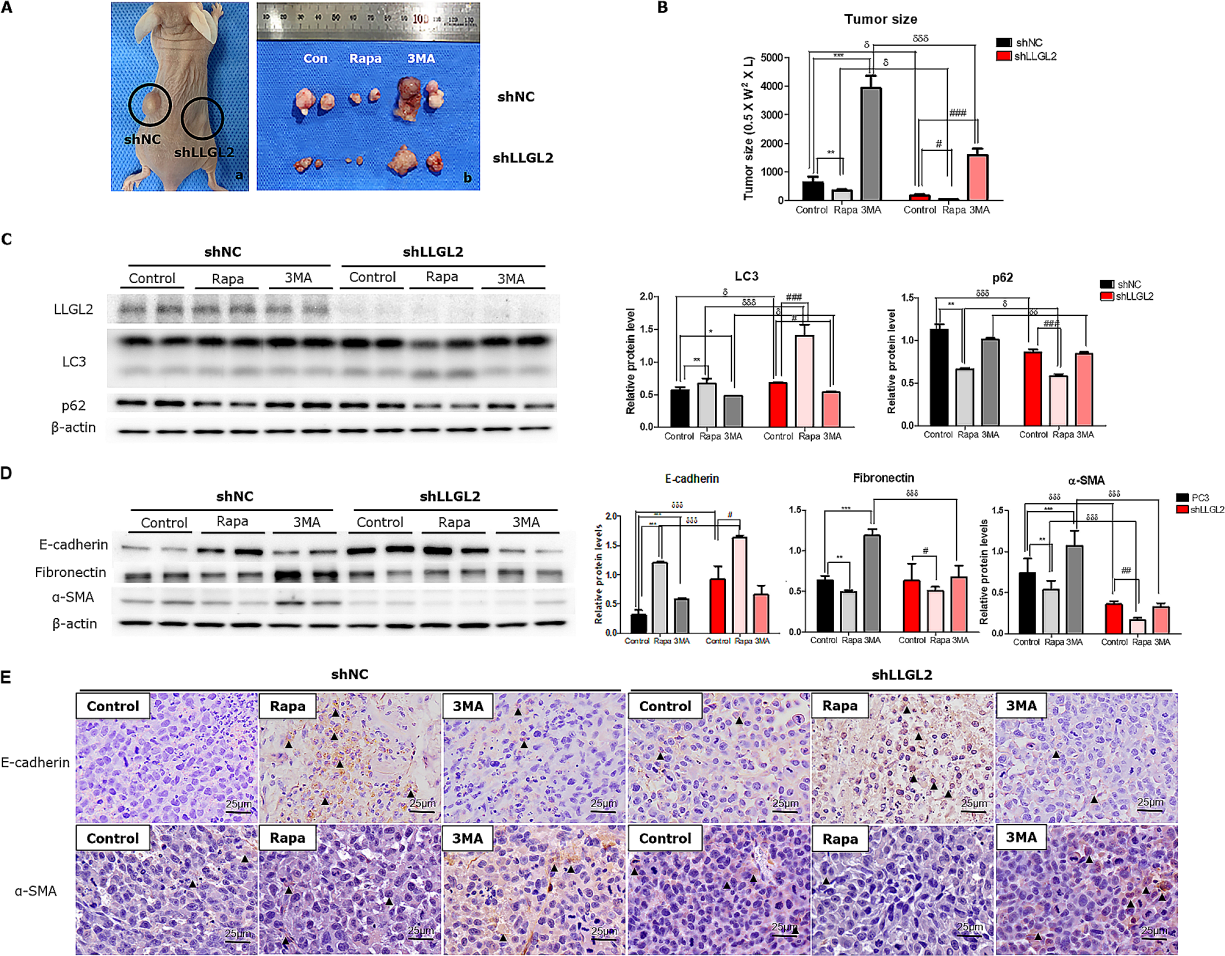
LLGL2 knockdown inhibits xenograft tumorS through autophagy in nude mice (*n* = 5/ group). Mice were inoculated subcutaneously into the right (shLLGL2) or left (shNC) flanks. For pharmacological changes in autophagy, mice were intraperitoneally treated with rapamycin (Rapa, 2.5 mg/kg/d) or 3-methyladenine (3MA, 30 mg/kg/d) when tumor size reached 100 mm^3^. (**A**) Representative images of the tumor obtained from each group. (**B**) Tumor size in each group was assessed by calipers and calculated as the length × width × width × 0.5. (**C**) Protein levels of LLGL2, LC3, and p62 were examined using western blotting in shNC or shLLGL2 tumor treated with or without Rapa or 3MA (left). Relative band intensities were analyzed using CS Analyzer (right). (**D**) The protein levels of E-cadherin, fibronectin, and α-SMA were examined using western blotting in shNC or shLLGL2 tumor treated with or without Rapa or 3MA (left). Relative band intensities were analyzed using CS Analyzer (Right). (**E**) Immunohistochemical staining of E-cadherin and α-SMA in tumor tissues from the mice. Scale bar = 25 μm. Error bar, mean ± SD. ^*^p < 0.05, ^**^p < 0.01, ^***^p < 0.001, vs. shNC-control, ^#^*p* < 0.05, ^##^*p* < 0.01, ^###^*p* < 0.001 vs. shLLGL2-control, and ^δ^*p* < 0.05, ^δδ^*p* < 0.01, and ^δδδ^*p* < 0.001 vs. respective control

To determine the role of LLGL2 in vivo, xenograft animal models were established by injecting stable PC3 cells with lentiviruses expressing shLLGL2 or shNC. The efficiency of infection and LLGL2 knockdown was confirmed using western blotting (Fig. 5C). To detect the effect of autophagy on EMT, we induced autophagy activation or impairment by intraperitoneal injection of rapamycin or 3MA. In the xenograft model, the tumor volume in the shLLGL2 group was lower than that in the shNC group. In particular, the tumor volume was significantly reduced in both Rapa-treated shNC and shLLGL2 groups compared to that in the respective controls. In contrast, 3MA administration significantly increased the tumor volume compared to the respective control volumes (Fig. 5A and B).

Figure 5C shows the autophagy-related markers obtained by measuring LC3 and p62 levels. The expression of LC3 was significantly increased in the tumors of mice in the shLLGL2 group compared to that in the shNC group. In contrast, p62 levels were reduced in the tumors of mice in the shLLGL2 group, indicating the activation of autophagy (Fig. 5C).

The protein level of E-cadherin was significantly increased (*p* < 0.001) whereas those of fibronectin and α-SMA were downregulated in the tumors of mice in the shLLGL2 group compared to those in the shNC group. Immunostaining results also showed that the E-cadherin positive area was increased in the tumors of mice in the shLLGL2 group compared to those in the shNC group. However, the positive-area of α-SMA was reduced in the tumors of mice in the shLLGL2 group compared to that in those in the shNC group. After rapamycin administration, EMT was attenuated, with an increase in epithelial marker and a decreases in mesenchymal markers. Conversely, 3MA administration worsened EMT progression by decreasing the expression of epithelial marker and increasing that of mesenchymal markers (Fig. 5D and E). In summary, these results indicate that silencing LLGL2 inhibit the EMT by inducing autophagy.

## Discussion

Emerging evidence has shown that LLGL2 contributes to tumorigenesis and progression both in vitro and in vivo [4, 5, 15, 16]. However, whether and how LLGL2 expression regulates the PCa progression remain unclear. In this study, we first discovered the role of LLGL2 in PCa both in vitro and in vivo. In human PCa tissues, the expression of LLGL2 was higher in human PCa tissues than in non-cancerous tissues. LLGL2 knockdown suppressed the invasion and migration of PC3 cells by attenuating EMT through upregulation of E-cadherin and downregulation of fibronectin and α-SMA, whereas overexpression of LLGL2 reversed the effect of LLGL2 knockdown on the EMT of PC3 cells. We further found that knockdown of LLGL2 mediated EMT suppression through autophagy induction, as inhibition or activation of autophagy could regulate EMT, however, overexpression of LLGL2 attenuated autophagy and promoted the EMT in PCa. These results suggest a probable mechanism for LLGL2 in PCa progression.

LLGL2 is a human homolog of Lgl, that plays an has important role in establishing the epithelial phenotype, regulating cell polarity, and orchestrating asymmetrical division in Drosophila [1]. In humans, LLGL2 has been implicated in the progression of various cancers, including breast, hepatic, and pancreatic cancer [4, 5, 17]. We recently demonstrated that LLGL2 is involved in autophagy regulation in BPH-1 prostate epithelial cells. LLGL2 activated the autophagy resulting in reduced proliferation of prostate cells [13]. Based on our results obtained BPH-1 cells, we examined the role of LLGL2 in PCa. In the patients with PCa, LLGL2 is highly expressed with defective autophagy compared to non-cancerous patients. From this perspective, we postulated that the role of LLGL2 in PCa involves the regulation of autophagy. Our results showed that the downregulation of LLGL2 significantly induced autophagy. In particular, knockdown of LLGL2 upregulated Vps34 and ATG14L, which are related to autophagosome formation, and we confirmed the increased expression of Vps34 and ATGL14 through immunofluorescence. Induction of autophagy flux by increasing autophagosome formation under siLLGL2 treatment was observed in LC3 puncta. Thus, the absence of LLGL2 induces a high autophagic flux to maintain homeostasis. This hypothesis was supported by the results of LLGL2 overexpression. In particular, ATG14L was downregulated when LLGL2 was overexpressed, accompanied by the inhibition of autophagic flux. Zheng et al. have reported that apicobasal polarity-related proteins play a role in autophagy. They showed that depletion of Cdc42 which controls cell polarity results in hyperplasia of intestinal crypts by regulating autophagy [18]. Similarly, our results showed that autophagy induced by the knockdown of LLGL2 in prostate cancer cells was due to the loss of polarity when the LLGL2 was knockdown. Therefore, LLGL2 may play a role in regulating of autophagy in prostate cancer cells.

Autophagy is a conserved self-degradation process that maintain homeostasis under basal conditions during cellular stress [19, 20]. One of the most prominent characteristics of autophagy in cancer cells is that it is highly complex and dynamic, but not immutable. Under normal conditions, basal autophagy acts as a tumor suppressor to maintain genomic stability. When a tumor is established, impaired autophagy contributes to cancer cell survival in the tumor microenvironment and promotes tumor growth and EMT [21].

EMT is an important biological process by which cancer cells acquire robust migratory and invasive capabilities, resulting in advanced cancer metastasis [22]. This process can disrupt cell-to-cell or cell-to-extracellular matrix adhesion in the polar epithelial lining, thereby promoting transition to mesenchymal cells [23]. E-cadherin is enriched in epithelial cells, which differ in phenotype and function from mesenchymal cells, that express high levels of N-cadherin, fibronectin and α-SMA [24, 25]. EMT is closely associated with invasion and migration of prostate cancer cells [26]. In our study, LLGL2 knockdown upregulated epithelial markers and downregulated mesenchymal markers, indicating that the EMT was inhibited. Furthermore, LLGL2 knockdown inhibited metastasis, accompanied by decreased invasion, migration, and wound closure in PCa cells. In contrast, overexpression of LLGL2 produced results opposite to those of siLLGL2, indicating that the presence of LLGL2 could increase metastasis and promote EMT.

In the early stages of metastasis, autophagy can reduce the invasion and migration of cancer cell from origin with antimetastatic effects. Several studies have shown that triggering autophagy suppresses migration, invasion and survival of PCa cells [27, 28]. Additionally, deletion of autophagy-related proteins can increase the invasion and migration through EMT progression [29]. Therefore, we investigated the role of autophagy in EMT progression following treatment with an autophagy inducer (Rapa) and inhibitor (3MA). Rapa treatment induced autophagy and inhibited EMT by upregulating E-cadherin and downregulating fibronectin and α-SMA compared with the control. However, when autophagy was blocked by 3MA, the results were contrary to those obtained after Rapa treatment. These results were confirmed by fluorescence staining of E-cadherin and fibronectin double staining. Our results showed that LLGL2 knockdown markedly induced autophagy resulting in EMT inhibition in PCa cells, similar to effects of Rapa treatment. Moreover, LLGL2 overexpression attenuated autophagy by promoting EMT, similar to 3MA treatment. Lv et al. reported that the activation of autophagy is closely related to repression of EMT through Snail and Twist in breast cancer [30]. In PCa, inhibition of serum- and glucocorticoid protein kinase (SGK1) induces autophagy by reversing EMT [31]. These results support our finding that LLGL plays an important role in EMT by regulating autophagy.

## Conclusions

In conclusion, we have demonstrated the role of LLGL2 expression in autophagy during PCa progression. Defective expression of LLGL2 reduces tumor size in prostate cancer and attenuates of EMT by upregulating autophagic flux. To the best of our knowledge, this is the first study to connect LLGL2 with the EMT and autophagy machinery. In summary, our results suggest that LLGL2 may be effective target for EMT in PCa through the regulation of autophagy.

### Electronic supplementary material

Below is the link to the electronic supplementary material.

Supplementary Material 2

Supplementary Material 2

Supplementary Material 3

## Data Availability

The datasets generated and analyzed during the current study are available from the corresponding authors on request.

## Abbreviations

3MA: Methyladenine
ATG14L: Autophagy-related genes 14 L
EMT: epithelial-mesenchymal transition
LC3: microtubule associated protein- 1 light chain 3α
LLGL: human homologues of Lethal giant larvae (Lgl)
PCNA: proliferating cell nuclear antigen
PCa: Prostate cancer
