# Supplementary figures and images for: Novel role of LLGL2 silencing in autophagy: reversing epithelial-mesenchymal transition in prostate cancer
Source: Biol Res. 2024 May 8;57:25. doi: 10.1186/s40659-024-00499-w (PMC11077766; doi:10.1186/s40659-024-00499-w)

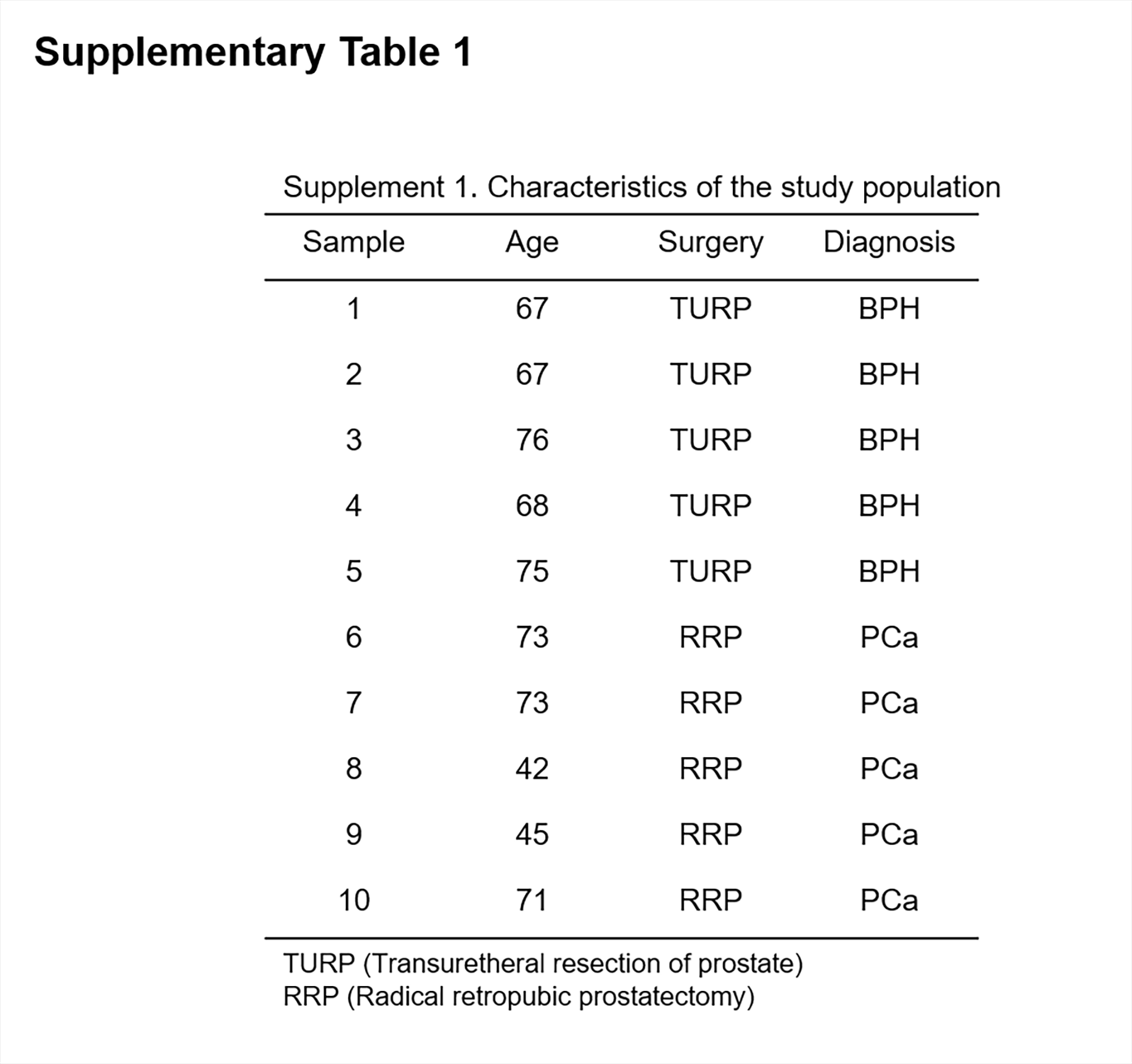

Supplement: Supplementary file 1 — Supplementary Material 2 [file 40659_2024_499_MOESM1_ESM.tif]

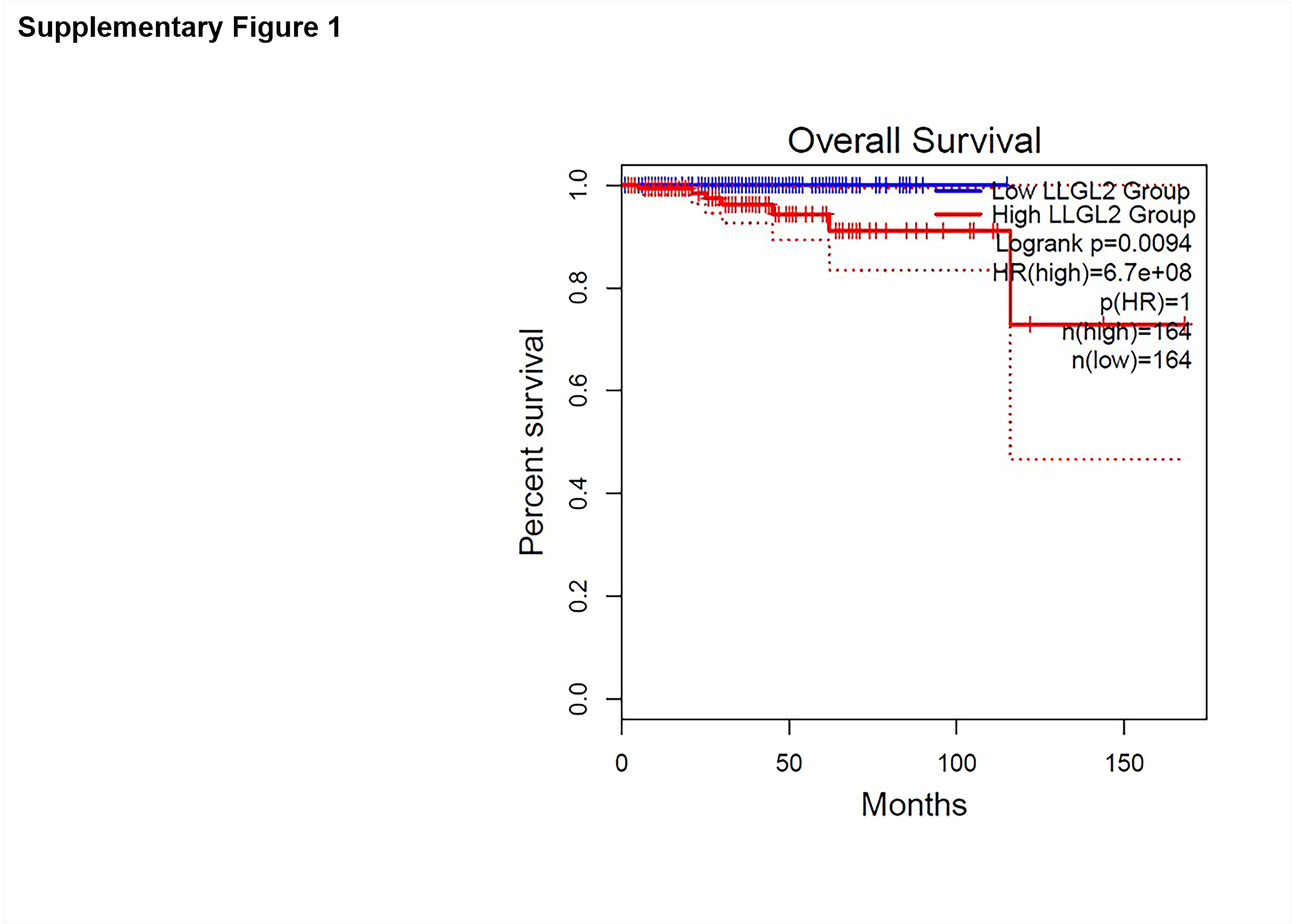

Supplement: Supplementary file 2 — Supplementary Material 2 [file 40659_2024_499_MOESM2_ESM.tif]

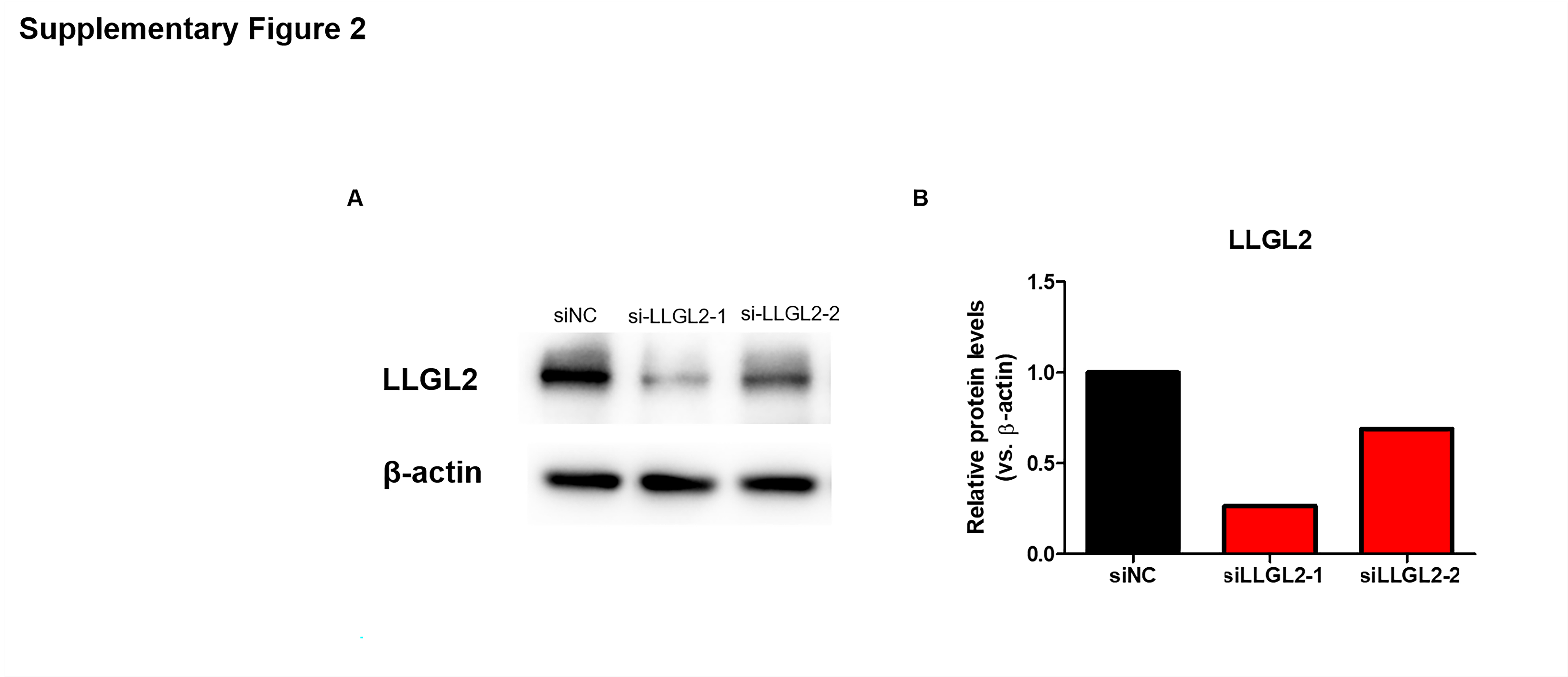

Supplement: Supplementary file 3 — Supplementary Material 3 [file 40659_2024_499_MOESM3_ESM.tif]
